# Changes in the Retinal Microvasculature Measured Using Optical Coherence Tomography Angiography According to Age

**DOI:** 10.3390/jcm9030883

**Published:** 2020-03-24

**Authors:** Seung Hun Park, Heeyoon Cho, Sun Jin Hwang, Beomseo Jeon, Mincheol Seong, Hosuck Yeom, Min Ho Kang, Han Woong Lim, Yong Un Shin

**Affiliations:** Department of Ophthalmology, Hanyang University College of Medicine, Seoul 04763, Korea

**Keywords:** optical coherence tomography angiography, age, retinal capillary vascular density, choriocapillaris vascular density, swept-source optical coherence tomography

## Abstract

In this cross-sectional study, we examined age-related changes in the retinal vessels of 100 healthy participants, aged from 5 to 80 years, and divided into four groups (G1, under 20 years of age; G2, from 20 to 39 years of age; G3, from 40 to 59 years of age; G4, age 60 years or older). All subjects underwent swept-source optical coherence tomography (SS-OCT) and OCT angiography (OCTA). The vascular density (VD) of the superficial (SCP) and deep capillary plexus (DCP), and choriocapillaris (CCP) were measured using OCTA. The vascular density of each capillary layer, foveal avascular zone (FAZ) area, ganglion cell-inner plexiform layer (GC-IPL) thickness, retinal thickness (RT), and choroidal thickness (CT) were compared between age groups. Most OCT variables were correlated with OCTA variables. The FAZ area; VD of the SCP, DCP, and CCP; GC-IPL thickness; RT; and CT showed significant difference (*p* < 0.001) between G1 + G2 and G3 + G4, except for central GC-IPL thickness (*p* = 0.14) and central RT (*p* = 0.25). Density of the retinal capillary vasculature reduced and FAZ area increased after age 40, which represents the onset of middle age.

## 1. Introduction

Retinal vascular diseases, such as diabetic retinopathy (DR) and retinal vein occlusion (RVO), are by far the leading causes of vision loss in the working population in developed countries, and their prevalence is seen to increase with age [1]. There were approximately 7.7 million DR patients in the United States in 2010 and 16.4 million RVO patients worldwide in 2010 [2]. As the socioeconomic burden of DR and RVO is directly related to their severity, the possible rise in prevalence over the next 30 years might cause further issues in the future [3]. 

There have been many efforts to demonstrate the vascular features of the retinal capillaries. Some studies have used the histological examination as the gold standard approach for measuring the vascular density of retinal capillaries [4,5,6]. Others have used fundus photography, confocal scanning lasers [6,7], or fluorescein angiography [6]. Imaging of the retina using fluorescein angiography (FA) has been the primary imaging method for visualizing retinal vessels since its emergence in 1961 [8]. However, FA is invasive, time-consuming in nature, and incapable of evaluating the vascular density (VD) of specific layers in the retina. 

Optical coherence tomography angiography (OCTA) provides a new and noninvasive method for imaging each retinal capillary layer, including the superficial capillary plexus (SCP), deep capillary plexus (DCP), choriocapillaris (CCP), and foveal avascular zone (FAZ) [9]. Recent studies proved that the calculation of the macular vascular density using OCTA is reproducible and noninvasive [10,11,12,13,14,15]. 

Although there have been studies of VD and FAZ area in adults [13] and children [16] in Asian populations, most studies were on Western populations [12,14,17,18,19,20] and all used spectral-domain OCTA (SD-OCTA). Swept-source OCT (SS-OCT) provides deeper penetration of the laser beam [21], which allows detailed images and better visualization of the choroid, permitting better calculation of VD and choroidal thickness (CT). Recently, there was a study on VD and FAZ measured using SS-OCTA in a Japanese population but not across a range of age groups [22]. 

Therefore, we performed this study to measure the VD of each retinal layer (SCP, DCP, and CCP) and FAZ area, in addition to some other OCT variables, using SS-OCTA, and to examine their correlation with age and compare parameters between several age groups to elucidate when changes in retinal vessels and retinal structures occur.

## 2. Methods

### 2.1. Study Population 

This retrospective study was approved by the Institutional Review Board (IRB) of Hanyang University Guri Hospital, Gyunggi-do, Korea, and all research conducted adhered to the tenets of the Declaration of Helsinki (IRB no.2019-11-033). The IRB waived the need for consent, and data were accessed anonymously.

This study contained 100 healthy participants, aged from 5 to 80 years, who visited our outpatient clinic for an annual healthcare checkup and had undergone SS-OCT and OCTA in Hanyang University Guri Hospital, Korea. Participants were enrolled in this study after comprehensive ophthalmologic examinations including slit-lamp microscopy, intraocular pressure, and automated refractometry. Participants with systemic vascular diseases such as hypertension, diabetes, and connective tissue diseases; severe media opacity leading to an inability to obtain clear OCT images; refractive error of >+3.0 D or <−3.0 D; or pathologic ocular conditions including uveitis, age-related macular degeneration, glaucoma, history of ocular trauma, and prior ocular surgery were excluded from this study. 

### 2.2. Study Design 

All participants underwent SS-OCT and OCTA (DRI OCT-1 Triton^®^, Topcon Corporation, Tokyo, Japan). The SS-OCT used a tunable light source centered at 1050 nm. It applied an eye-tracking system during acquisition and proprietary ratio analysis (OCTARA) for angiographic processing. The right eye was chosen to rule out selection bias. An angiography scan covering a 3 × 3 mm area of the macula was performed and analyzed using automated vessel density calculator software (Version 1.22.13993) recently updated by Topcon.

OCTA images with significant artifacts and decreased image quality were excluded, including those with (1) a quality signal strength index <40, (2) definite residual motion artifacts, (3) indiscrete segmentation of each retinal layers or slabs, (4) poor centering, or (5) signal loss due to blinking. 

The SCP was defined as the layer starting with the inner limiting membrane (ILM) and selecting an acceptable thickness that contains the ganglion cell layer, between 3 µm below the ILM and 15 µm below the inner border of the inner plexiform layer (IPL). The DCP comprised the capillaries in the layer between 15 µm below the inner border of the IPL and 70 µm below the inner border of IPL. The CCP consisted of the capillaries in the layer from the inner border of Bruch’s membrane (BM) to 10.4 µm below it. 

Vascular density (total vessel area/total measured area%) was defined as the proportion of the area filled by vessel lumens in the selected area. The percentage vessel density was automatically generated by a built-in software and was calculated in five divisions (superior, temporal, inferior, nasal, and central) based on a modified Early Treatment Diabetic Retinopathy Study (ETDRS) approach where the center of the fovea was automatically settled. The inner and outer rings with a diameter of 1 and 3 mm around the fovea were respectively considered for evaluation. In the CCP, we used the inverse transformation to compensate for choriocapillaris signal attenuation due to structural changes in the RPE/basement membrane (BM) complex. A simple multiplication between the inverted en-face structural CCP image and the en-face flow CCP image was performed as described by Zhang et al. [23]. Using the compensated CCP image, we performed automatic local thresholding to obviate small regional variations, as described by Spaide [24] using ImageJ ver. 1.8.0 (https://imagej.nih.gov/ij/index.html). Thereafter, using this thresholded binarized CCP image, the percentage of flow deficits (FD%, the percentage area with flow deficit in each analyzed area) in each of the central and parafoveal regions was calculated using ImageJ. Using FD%, we calculated the VD of the CCP.

We used the superficial capillary plexus layer to evaluate the FAZ area. FAZ area was calculated manually by two examiners (Y.U.S. and S.J.H.) using a built-in software tool; the average of their measurements was used in this study. Ganglion cell-inner plexiform layer (GC-IPL) thickness was measured using automated software in Topcon DRI-OCT-1 also following the ETDRS grid. We calculated the GC-IPL thickness in the superior, temporal, inferior, and nasal sectors of the ETDRS grid using automated segmentation with manual segmentation error correction. As SS-OCT penetrates the deep tissue well, retinal thickness (RT) and choroidal thickness (CT) were measured automatically utilizing the image that aligned to the center of fovea correctly. In the case of segmentation artifacts, the manual correction was performed by two examiners, and the average value was used.

### 2.3. Subgroup Analysis 

In place of assessing age-related data, subjects were divided into four age groups; Group 1 (G1) included those aged 5 to 19 years of age, Group 2 (G2) included those aged 20 to 39 years, Group 3 (G3) included those aged 40 to 59 years, and Group 4 (G4) included subjects who were older than 60 years old. We compared the youngest group with the other older groups (G1 and G2, G1 and G3, and G1 and G4) to identify vascular density changes in the retina and choroid with age. G1 + G2 and G3 + G4 were then compared to identify whether the change in the retinal vascular system occurs after middle age, which had been defined in this study as starting at 40 years of age. 

### 2.4. Statistical Analysis 

One-way analysis of variance (ANOVA) was used to test for differences between groups, and Tukey post hoc tests were used to test for differences between G1 and the other groups. The independent t-test was used to compare G1 + G2 and G3 + G4. Age-related changes in FAZ area, VD of the SCP, VD of the DCP, VD of the CCP, GC-IPL thickness, RT, and CT were evaluated using Pearson’s correlation. Pearson’s correlation was also used to assess the relationship between structural OCT parameters (GC-IPL thickness, RT, and CT) and OCTA parameters (VD of the SCP, DCP, and CCP). All statistical analyses were performed using SPSS software, Version 22.0 (IBM Corp., Armonk, NY, USA). All data are presented as mean ± standard deviation, and statistical significance was set at a *p*-value of 0.05. 

## 3. Results

We analyzed 100 healthy participants and their demographic information is listed in Table 1. The mean age of all participants was 35.90 ± 24.23 years and the mean spherical equivalent was 0.34 ± 1.23 diopters. Fifty male and 50 female participants were included. The mean age of G1, G2, G3, and G4 was 10.19 ± 3.38, 29.2 ± 6.53, 52.35 ± 6.02, and 69.09 ± 5.76 years, respectively. Spherical equivalent of each group was −0.56 ± 1.21, −0.60 ± 1.10, −0.50 ± 1.28, and 0.41 ± 1.10 D. 

**Figure 1:**
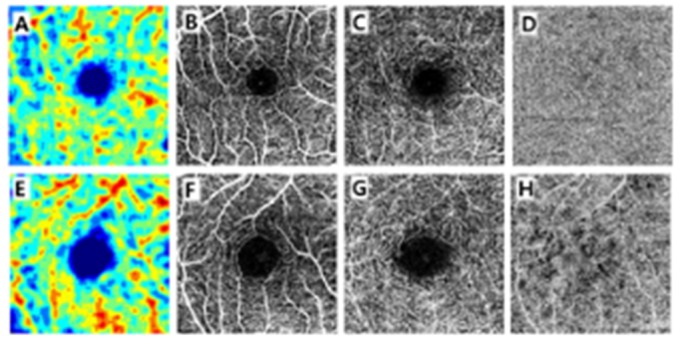
Representative OCTA images from participants of different ages. The top row shows OCTA images from a 14-year-old boy and the bottom row shows those from a 65-year-old man. The color-coded maps of the superficial capillary plexus of a 14-year old boy (**A**) and a 65-year-old man (**E**) show a difference in FAZ area, which is located in the center of the macula, and seen as a blue color. Furthermore, angiographic images of both subjects (**B,F**) demonstrated that a 65-year-old man had coarse superficial capillary density and this feature remained constant in the deep capillary plexus (**C,G**) and choriocapillaris (**D,H**).

**Figure 2:**
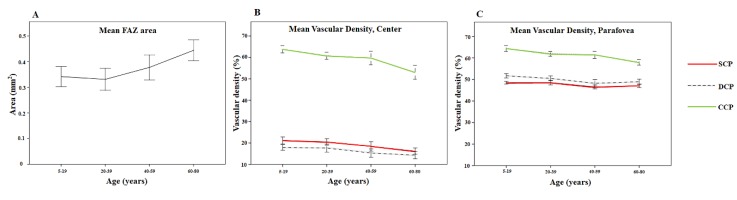
Line graph showing the relationship between age and FAZ area (mm^2^) (**A**), central mean vessel densities (%) (**B**), and parafoveal mean vessel densities (%) (**C**). Error bars represent one standard deviation.

Table 2 shows the mean measurements of the FAZ. The mean FAZ area in each group was 0.341 ± 0.116, 0.331 ± 0.091, 0.377 ± 0.104, and 0.443 ± 0.091 mm^2^ for G1, G2, G3, and G4, respectively. The FAZ area differed significantly between groups (*p* = 0.001). There was a statistically significant difference between G1 and G4 (*p* = 0.002). Figure 1 shows the difference in FAZ size. In Figure 2A, the age and FAZ area are shown as a line graph.

The mean VD according to ETDRS sectors and the four age groups among SCP, DCP, and CCP are shown in Table 3 and Figure 2B,C. Parafovea, here, represents the average of the superior, temporal, inferior, and nasal sectors of the outer ring in the ETDRS grid. There was a significant difference among groups regarding the center and parafovea of SCP, DCP, and CCP (all *p* < 0.001; ANOVA). According to post hoc analysis, comparing G1 with G2, there was no statistically significant difference in the VD of the SCP, DCP, and CCP. The parafoveal VD of the SCP, DCP, and CCP in G3 were lower than those in G1 (*p* = 0.03, *p* < 0.001, *p* = 0.014, respectively; tukey post hoc test) but the difference in the central VD of the SCP, DCP, and CCP were not statistically significant (*p* = 0.09, *p* = 0.096, and *p* = 0.064, respectively; tukey post hoc test). All VD parameters in G4 were lower than those in G1 (all *p* < 0.001; tukey post hoc test). 

Table 4 compares the retinal and choroidal thickness between age groups. All thickness parameters differed significantly between groups except for the central GC-IPL thickness (*p* = 0.098; ANOVA). The parafoveal GC-IPL thickness, parafoveal RT, and CT (both of the center and parafovea) in G4 were lower than those in G1 (*p* = 0.027, *p* = 0.001, *p* = 0.02, and *p* = 0.011, respectively; tukey post hoc test). There was no statistically significant difference in retinal and choroidal thickness between G1 and the other two groups.

The results of comparing G1 + G2 and G3 + G4 between younger adults and older adults over 40 years of age are shown in Table 5. Older adults had a larger FAZ area (0.412 ± 0.102 mm^2^) than younger adults‘ (0.338 ± 0.107 mm^2^) (*p* < 0.001). The VD at the level of the SCP, DCP, and CCP was lower in older adults (all *p* < 0.001). All thickness parameters were significantly lower in older adults except for the center GC-IPL thickness (*p* > 0.14) and center RT (*p* > 0.25). 

**Figure 3:**
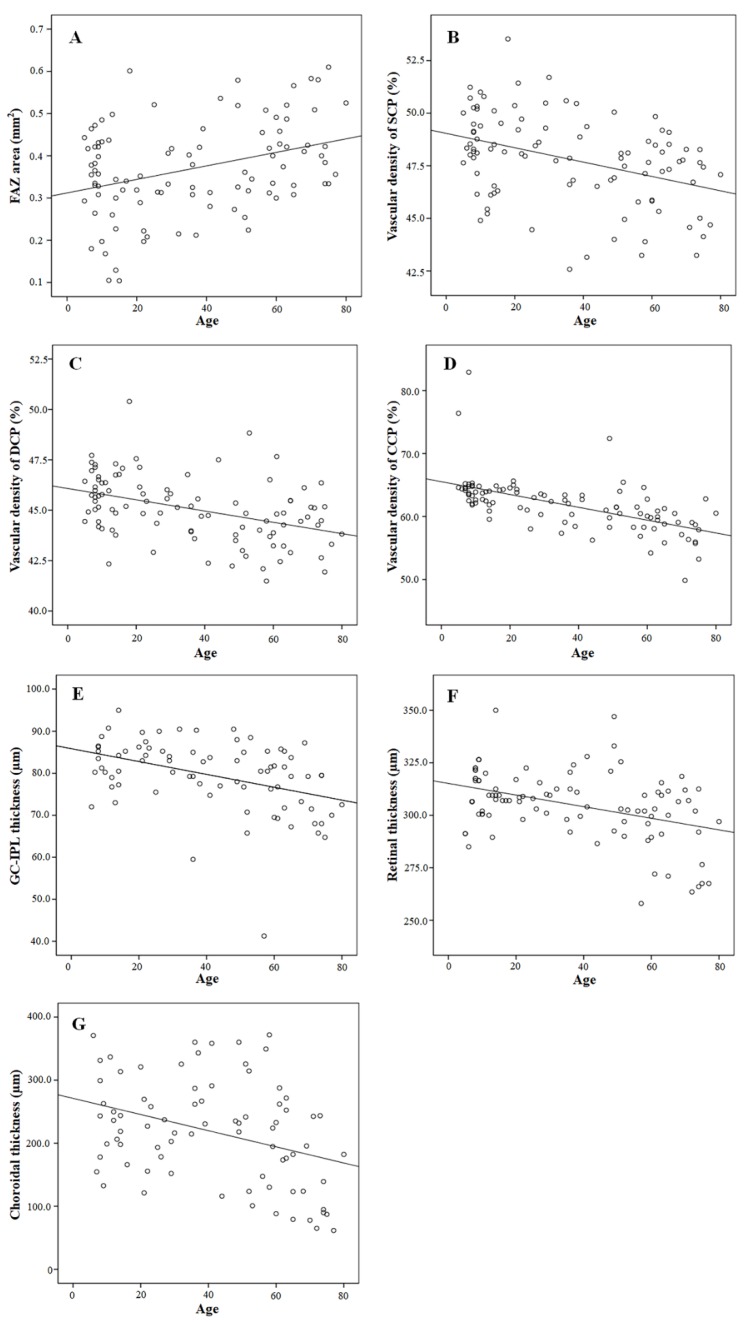
Correlation between age and other variables. The FAZ area was positively correlated with age (R = 0.350, *p* < 0.001) (**A**). (**B**–**G**) show correlations between age and OCTA and OCT variables of the parafoveal region of the ETDRS grid. The VD of the SCP (R = −0.388, *p* < 0.001) (**B**), VD of the DCP (R = −0.420, *p* < 0.001) (**C**), VD of the CCP (R = −0.590, *p* < 0.001) (**D**), GC-IPL thickness (R = −0.412, *p* < 0.001) (**E**), RT (R = −0.409, *p* < 0.001)(**F**), and CT (R = −0.354, *p* < 0.001) (**G**) were negatively correlated with age.

Figure 3 shows the correlation between age and OCTA and OCT variables. A positive correlation was found between age and FAZ area (Figure 3A, R = 0.350, *p* < 0.001). The parafoveal VD of the SCP (R = −0.388, *p* < 0.001), parafoveal VD of the DCP (R = −0.420, *p* < 0.001), parafoveal VD of the CCP (R = −0.590, *p* < 0.001), parafoveal GC-IPL thickness (R = −0.412, *p* < 0.001), parafoveal RT (R = −0.409, *p* < 0.001), and parafoveal CT (R = −0.354, *p* = 0.001) were negatively correlated with age (Figure 3B–G). There were also a statistically significant correlation between age and the center VD of the SCP (R = −0.421, *p* < 0.001), VD of the DCP (R = −0.364, *p* < 0.001), VD of the CCP (R = −0.564, *p* < 0.001), central GC-IPL thickness (R = −0.229, *p* = 0.043), and central CT (R = −0.313, *p* = 0.005).

In Pearson’s correlation analysis, FAZ area was significantly correlated with the center part of the SCP VD, DCP VD, CCP VD, GC-IPL thickness, RT, and CT (Table 6). In the parafoveal region, CCP VD and all thickness parameters were significantly correlated with FAZ area.

In the central region of the ETDRS grid, GC-IPL thickness was significantly correlated with the VD of the SCP (R = 0.630, *p* < 0.001) and DCP (R = 0.647, *p* < 0.001). RT was significantly correlated with the VD of the SCP (R = 0.512, *p* < 0.001) and DCP (R = −0.452, *p* < 0.001). CT was also significantly correlated with the VD of the CCP (R = 0.285, *p* = 0.011). At the parafoveal region, there were significant correlations between the GC-IPL thickness and VD of the retinal capillary plexus (R = 0.456, *p* < 0.001 in the SCP; R = 0.282, *p* = 0.012 in the DCP). RT was correlated with the VD of the SCP (R = 0.260, *p* = 0.01). CT was significantly correlated with the VD of the CCP (R = 0.302, *p* = 0.007).

## 4. Discussion

In this study, changes in FAZ size, the VD of the retinal capillary plexus and choriocapillaris, and thickness of the retina and choroid were studied. We found that the retinal and choroidal capillary densities and thickness of the retina and choroid tend to decrease with age, especially after 40 years of age. After middle age, FAZ was enlarged, parafoveal VD decreased, and the thickness was partially reduced. This is the first study that statistically evaluated age-related changes in the chorioretinal capillary plexus across various age groups using SS-OCTA.

Several previous studies have tried to establish normative data of retinal capillary vascular densities and FAZ area and demonstrate a relationship with age. Shahlaee et al. [10] reported a mean SCP VD of 32% and 46% in the center and parafovea, respectively, and a mean DCP VD of 27% and 52% in the center and parafovea, respectively. They calculated the central VD in the SCP and DCP in a circular region with a 1.2 mm diameter and parafoveal region defined as a ring 91 pixels wide surrounding the foveal region using SD-OCT. Despite different definitions of the central and parafoveal area, we believe that our results are comparable. Considering the smaller defined area, central VD in this study may be smaller than that in the previous study. Coscas et al. [12] divided patients into three groups according to their age and compared the VD of the retinal capillary plexus based on the ETDRS chart, which had inner and outer rings with a diameter of 1 and 2.5 mm around the fovea, using SD-OCT. They reported a central SCP VD, parafoveal SCP VD, central DCP VD, parafoveal DCP VD, and FAZ area of 31%, 54%, 30%, 60%, and 0.28 mm^2^, respectively. In another study, the VD in the central SCP and DCP was reported to be 19% and 15%, respectively [22], similar to our findings of 19% and 16%. VD in the parafoveal SCP and DCP (48% and 50%, respectively) were also like ours (47% and 50%). It is thought that similar results were obtained with the use of SS-OCT and ETDRS charts for East Asians. Iafe et al. [14] used SD-OCT and OCTA to report a decrease in the VD of the SCP and DCP with increasing age. They measured a mean FAZ area of 0.289 mm^2^. The results of the present study correspond with those od earlier studies that reported that age is negatively correlated with the VD of the retinal capillary plexus and positively correlated with the FAZ area. Gadde et al. [13] and Yu et al. [15], however, suggested that the VD of the retinal microvasculature was not apparently correlated with age. It is thought that many of the participants were younger than 25 years old in those studies.

The retinal structural changes according to age have been evaluated by several studies using OCT [19,25,26]. Yantao et al. [19] recruited participants aged from 18 to 82 years and divided them into four groups based on age. They found that age was negatively related to retinal vessel density and inner retinal layer thickness, and positively related to the outer plexiform layer (OPL) thickness and photoreceptor (PR) thickness. In one previous study, the longitudinal analyses revealed that overall GC thickness decreased by 0.25 μm per year and the cross-sectional analyses showed the GC thickness was 0.17 μm thinner per year after the age of 40 [26]. Our study revealed an age-related decrease in GC-IPL thickness. RT also decreased with age, but there was no statistical significance in central RT. The results of the present study are consistent with previous data, suggesting that inner retinal thickness, including GC-IPL, is negatively related to age. It is still unclear why the central retinal thickness remains unchanged while retinal microvascular density, GC-IPL thickness, and CT decreased significantly with age. Hence, further study is needed to demonstrate why this would occur.

The relationship between retinal capillary VD and retinal thickness in healthy participants has been reported previously. Jian et al. found that retinal perfusion was correlated with the inner retinal thickness but not with full retinal thickness [15]. Zhang et al., however, found that both foveal and parafoveal retinal thicknesses were significantly positively correlated with the foveal VD at the level of the SCP and DCP. [16] Similar results were found in our study. The central and parafoveal VD were significantly positively correlated with the central and parafoveal GC-IPL and RT at the level of the SCP and DCP. Unlike previous studies, we also investigated the relationship between the VD of the CCP and chorioretinal thickness. The central VD of the CCP was only related to the central CT, but the parafoveal VD of the CCP was correlated with all thickness parameters. Although CCP does not represent the entire choroidal flow, in this study, the VD of the CCP and choroidal thickness were correlated, suggesting that choroidal blood flow and choroidal thickness are related. 

Several studies tried to present normative data from children. Zhang et al. [16] analyzed 75 eyes in healthy children, aged from 8 to 16 years. They used the ETDRS grid to demonstrate the vascular density of the SCP (56.61%), DCP (63.66%), and CCP (66.29%). Our study found a lower vessel density in the SCP (48.54 %), DCP (51.72 %), and CCP (64.44%). The results of the present study correspond with this study that reported that the FAZ area is 0.290 mm^2^, central RT is 238.79 µm, and CT is 260.95 µm. Yilmaz et al. [20] compared 30 amblyopic eyes with 15 healthy normal control eyes. They reported a FAZ area of 0.280 mm^2^ in normal healthy controls. Our study found a FAZ area of 0.341 mm^2^ in G1. This might be due to differences in the number of samples, differences in measuring OCT devices, or race of the examined subjects.

We also tried to identify the critical age with respect to changes in retinal vascular features. Many studies [27,28] found that most vascular diseases, such as cardiovascular, cerebrovascular, and retinal vascular diseases, occur more frequently after 40 years of age. In addition, our results showed that the mean FAZ area starts to increase between G2 and G3 and just keeps on rising at a similar rate between G3 and G4. This was why our study selected the age of 40 years when grouping participants based on age. We were interested in elucidating whether there would be any significant change before age 40; consequently, we first made two groups, one that included young children aged 5 to 19, and a young adult group aged 20 to 40. As these two groups showed no difference in the FAZ area, capillary vascular densities in the SCP, DCP, GC-IPL thickness, RT, and CT, we advanced our study to divide participants into two groups based on a cut-off age of 40. Capillary densities in all retinal layers, choriocapillaris, parafoveal GC-IPL thickness, parafoveal retinal thickness, and CT in the younger group aged less than 40 were larger than those in the older group aged more than 40. However, the FAZ area was lower in the younger group than in the older group. The variables we measured and compared between groups showed age-related changes and were different before and after age 40. Hence, an assumption can be made that age 40 is a critical age inducing age-related changes in retinal microvasculature and retinal structure. 

Our study has some limitations. First, it was a retrospective design and had a relatively small sample size. Even though we covered all age groups from 5 to 80 years, it would be better to enroll more patients to strengthen our conclusions. However, recent OCTA studies on the VD of the retina in healthy eyes also used similar sample sizes. Sato et al. studied 145 eyes in healthy Japanese participants to evaluate SCP VD, DCP VD, and FAZ area using SS-OCTA [22]. Iafe et al. included 113 eyes [14] and Wei et al. enrolled 64 eyes to investigate age-related alterations in retinal VD. [19] Therefore, this study, despite the small sample size of each age group, is still worth comparing with other studies. Second, the axial length of participants was not obtained and various aspects of refraction, such as severe myopia and hyperopia, were not included. Danuta et al. [29] found that there were differences in superficial capillary plexus density and the FAZ area between corrected and uncorrected eyes for axial length. However, they analyzed subjects with a mean spherical equivalent error of −1.65 D (−8.00 D to +4.88 D), while participants in our study had a mean spherical equivalent error of −0.32 D (−2.88 D to +3 D). Even though the participants in our study had little variation among them, more precise measurement calibrations for axial length would be needed in future studies. Third, our study used a 3 × 3 mm OCTA scan, which only covered the parafoveal area. With the advances in built-in software, we expect to measure the vascular density of the peripheral retinal area in the future. Fourth, an OCTA also has certain inherent issues. OCTA has several artifacts, including motion, blinking, and centering artifacts, which could lead to an error in calculating vessel density. We performed manual correction to reduce segmentation errors as much as possible. Unlike previous studies, we used the post-processing of CCP images to compensate for choriocapillaris signal attenuation. Using this method, CCP VD can be obtained more accurately by reducing errors caused by attenuation [23]. Fifth, in this study, the male to female ratio in the G4 group is somewhat different compared to other age groups. Niestrata-Ortiz et al. [30] reported that there were differences in FAZ area and chorioretinal thickness by gender, but Sato et al. [22] reported that there were no gender differences in vascular density of retinal capillary plexus. Care should be taken in interpreting the results for the G4 group, as differences in gender-dependent VD remained to be confirmed. 

Despite the limitations above, we also have several strengths. First, we covered nearly all ages from 5 to 80 years and identified vascular and structural changes in the retina and choroid that occurred with age. Second, SS-OCT was utilized, which has better imaging resolution than SD-OCT because of its longer wavelength. The 1050 nm wavelength allows better visualization of deeper layers, such as the CCP, and the higher number of scans made more accurate measurement possible. SS-OCT has a more rapid scanning speed when compared with that of SD-OCT, and we could easily obtain OCT data from uncooperative subjects, such as young children and the elderly.

In conclusion, our study suggests that changes in the retinal microvasculature occur during aging, and these changes happen, especially after age 40, which represents the onset of middle age. However, further researches are warranted on why the incidence of retinal vascular disease increases after middle age.

## Figures and Tables

**Table 1:** Demographic data from healthy participants in the study.

Variable	G1 (*n* = 36)	G2 (*n* = 20)	G3 (*n* = 22)	G4 (*n* = 22)	Total (*n* = 100)
Male/Female	15/21	10/10	10/12	15/7	50/50
Age (years)	10.19 ± 3.38	29.2 ± 6.53	52.35 ± 6.02	69.09 ± 5.76	35.90 ± 24.23
SE (D)	−0.56 ± 1.21	−0.60 ± 1.10	−0.50 ± 1.28	0.41 ± 1.10	−0.34 ± 1.23

G1, age 5 to 19 years; G2, age 20 to 39 years; G3, age 40 to 59 years; G4, age 60 to 80 years; SE; spherical equivalent.

**Table 2:** Foveal avascular zone (FAZ) area measurements by age group.

FAZ Area (mm^2^)					*p*-Value ^a^	*p*-Value ^b^		
G1	G2	G3	G4	Total		G1 vs. G2	G1 vs. G3	G1 vs. G4
0.341 ± 0.116	0.331 ± 0.091	0.377 ± 0.104	0.444 ± 0.091	0.370 ± 0.111	0.001	0.983	0.602	0.002

^a^*p*-values derived using one-way analysis of variance.; ^b^*p*-values derived using Tukey post hoc test. G1, age 5 to 19; G2, age 20 to 39; G3, age 40 to 59; G4, age 60 to 80. FAZ; foveal avascular zone.

**Table 3:** Vascular density measurements by age groups.

OCTA Parameter	Vascular Density (%)					*p*-Value ^a^	*p*-Value ^b^		
	G1	G2	G3	G4	Total		G1 vs. G2	G1 vs. G3	G1 vs. G4
**SCP**									
center	21.22 ± 4.86	20.53 ± 3.09	18.46 ± 4.65	16.20 ± 3.28	19.39 ± 4.58	<0.001	0.934	0.090	<0.001
parafovea	48.54 ± 1.92	48.56 ± 2.25	46.57 ± 1.97	47.08 ± 1.81	47.81 ± 2.13	<0.001	1.000	0.030	0.037
**DCP**									
center	17.94 ± 4.04	17.63 ± 3.97	15.33 ± 4.18	14.36 ± 3.80	16.54 ± 4.23	<0.001	0.992	0.096	0.007
parafovea	51.72 ± 2.89	50.53 ± 2.36	48.24 ± 3.68	48.96 ± 2.76	50.14 ± 3.22	<0.001	0.476	<0.001	0.004
**CCP**									
center	63.80 ± 5.06	60.76 ± 3.55	59.75 ± 6.73	53.02 ± 7.38	59.94 ± 6.99	<0.001	0.241	0.064	<0.001
parafovea	64.44 ± 4.04	61.98 ± 2.41	61.47 ± 3.58	57.95 ± 2.99	61.88 ± 4.18	<0.001	0.057	0.014	<0.001

^a^*p*-values derived using One-way analysis of variance; ^b^*p*-values derived using Tukey post hoc test; G1, age 5 to 19 years; G2, age 20 to 39 years; G3, age 40 to 59; G4, age 60 to 80 years; parafovea means the average of the superior, temporal, inferior, and nasal sectors of the outer ring of the Early Treatment Diabetic Retinopathy Study (ETDRS) grid; SCP; superficial capillary plexus, DCP; deep capillary plexus, CCP; choriocapillaris.

**Table 4:** Retinal and choroidal thickness by age group.

OCT Parameter	Thickness (μm)					*p*-Value ^a^	*p*-Value ^b^		
	G1	G2	G3	G4	Total		G1 vs. G2	G1 vs. G3	G1 vs. G4
**GCIPL thickness**									
center	45.72 ± 6.96	46.85 ± 5.85	46.20 ± 7.95	41.67 ± 7.84	45.05 ± 7.38	0.098	0.963	0.997	0.306
parafovea	92.54 ± 5.95	92.94 ± 6.99	87.91 ± 10.78	85.53 ± 8.46	89.53 ± 8.46	0.007	0.999	0.285	0.027
**RT**									
center	232.92 ± 16.66	237.30 ± 13.02	237.65 ± 19.12	222.59 ± 24.02	232.46 ± 19.04	0.031	0.892	0.794	0.171
parafovea	310.76 ± 12.63	308.93 ± 8.60	303.73 ± 19.74	294.23 ± 18.94	305.24 ± 16.37	0.001	0.973	0.355	0.001
**CT**									
center	244.78 ± 71.16	242.05 ± 71.29	243.40 ± 107.00	166.29 ± 71.89	222.87 ± 87.36	0.006	1	1	0.02
parafovea	241.14 ± 67.56	241.10 ± 65.43	232.77 ± 93.80	162.43 ± 75.38	218.09 ± 82.37	0.003	1	0.987	0.011

^a^*p*-values derived using one-way analysis of variance; ^b^*p*-values derived using Tukey post hoc test; G1, age 5 to 19 years; G2, age 20 to 39 years; G3, age 40 to 59 years; G4, age 60 to 80 years; parafovea means the average of the superior, temporal, inferior and nasal sectors of the outer ring of the ETDRS grid; GC-IPL; ganglion cell-inner plexiform layer, RT; retinal thickness, CT; choroidal thickness.

**Table 5:** Comparison between G1 + G2 and G3 + G4.

OCT & OCTA Parameter	G1 + G2	G3 + G4	*p*-Value
**FAZ (mm^2^)**	0.338 ± 0.107	0.412 ± 0.102	<0.001
**SCP VD (%)**			
center	20.98 ± 4.29	17.28 ± 4.10	<0.001
parafovea	48.55 ± 2.02	46.84 ± 1.88	<0.001
**DCP VD (%)**			
center	17.83 ± 3.98	14.82 ± 3.97	<0.001
parafovea	51.29 ± 2.75	48.62 ± 3.21	<0.001
**CCP VD (%)**			
center	62.72 ± 4.78	56.22 ± 7.77	<0.001
parafovea	63.56 ± 3.72	59.63 ± 3.70	<0.001
**GC-IPL thickness (µm)**			
center	46.32 ± 6.34	43.88 ± 8.13	0.143
parafovea	92.75 ± 6.44	86.54 ± 9.07	<0.001
**RT (µm)**			
center	234.48 ± 15.48	229.76 ± 22.86	0.252
parafovea	310.11 ± 11.31	298.75 ± 19.68	<0.001
**CT (µm)**			
center	243.34 ± 70.28	203.90 ± 97.70	0.042
parafovea	241.12 ± 65.54	196.74 ± 91.03	0.015

*p*-values derived using the independent t-test; G1 + G2, age 5 to 39 years; G3 + G4, age 40 to 80 years; Parafovea means the average of the superior, temporal, inferior, and nasal sectors of the outer ring of the ETDRS grid; FAZ; foveal avascular zone, VD; vascular density, SCP; superficial capillary plexus, DCP; deep capillary plexus, CCP; choriocapillaris, GC-IPL ganglion cell-inner plexiform layer, RT; retinal thickness, CT; choroidal thickness.

**Table 6:** Correlation analysis between FAZ area and other parameters.

OCT&OCTA Parameters	Center		Parafovea	
	R	*p*-Value	R	*p*-Value
SCP VD	−0.540	<0.001	−0.017	0.865
DCP VD	−0.428	<0.001	0.033	0.75
CCP VD	−0.214	0.034	−0.299	0.003
GC-IPL thickness	−0.471	<0.001	−0.331	0.003
RT	−0.351	<0.001	−0.346	0.015
CT	−0.231	0.04	−0.236	0.036

*p*-values derived using Pearson correlation analysis. FAZ; foveal avascular zone, VD; vascular density, SCP; superficial capillary plexus, DCP; deep capillary plexus, CCP; choriocapillaris, GC-IPL ganglion cell-inner plexiform layer, RT; retinal thickness, CT; choroidal thickness.
